# Assessment of Micronutrient Status in Critically Ill Children: Challenges and Opportunities

**DOI:** 10.3390/nu9111185

**Published:** 2017-10-28

**Authors:** Duy T. Dao, Lorenzo Anez-Bustillos, Bennet S. Cho, Zhilling Li, Mark Puder, Kathleen M. Gura

**Affiliations:** 1 Department of Surgery and Vascular Biology Program, Boston Children’s Hospital, 300 Longwood Avenue, Boston, MA 02115, USA; duy.dao@childrens.harvard.edu (D.T.D.); lorenzo.anez-bustillos@childrens.harvard.edu (L.A.-B.); bennet.cho@childrens.harvard.edu (B.S.C.); mark.puder@childrens.harvard.edu (M.P.); 2 Department of Pharmacy, Shanghai Children’s Hospital, Shanghai Jiao Tong University, 355 Luding Road, Shanghai 200062, China; lizl@shchildren.com.cn; 3 Department of Pharmacy and the Division of Gastroenterology and Nutrition, Boston Children’s Hospital, 300 Longwood Avenue, Boston, MA 02115, USA

**Keywords:** micronutrients, vitamins, minerals, critical illness, pediatric intensive care unit, neonatal intensive care unit

## Abstract

Micronutrients refer to a group of organic vitamins and inorganic trace elements that serve many functions in metabolism. Assessment of micronutrient status in critically ill children is challenging due to many complicating factors, such as evolving metabolic demands, immature organ function, and varying methods of feeding that affect nutritional dietary intake. Determination of micronutrient status, especially in children, usually relies on a combination of biomarkers, with only a few having been established as a gold standard. Almost all micronutrients display a decrease in their serum levels in critically ill children, resulting in an increased risk of deficiency in this setting. While vitamin D deficiency is a well-known phenomenon in critical illness and can predict a higher need for intensive care, serum concentrations of many trace elements such as iron, zinc, and selenium decrease as a result of tissue redistribution in response to systemic inflammation. Despite a decrease in their levels, supplementation of micronutrients during times of severe illness has not demonstrated clear benefits in either survival advantage or reduction of adverse outcomes. For many micronutrients, the lack of large and randomized studies remains a major hindrance to critically evaluating their status and clinical significance.

## 1. Introduction

Micronutrients refer to a group of organic vitamins and inorganic trace elements, all of which play a wide range of essential functions in maintaining the body’s homeostasis. They serve as co-factors for many important metabolic enzymes, regulate gene transcription, and power the body’s defense against oxidative stress [1]. Most micronutrients circulate in association with carrier proteins, the levels of which are reduced by the effects of acute inflammation and the body’s response to physiologic stress. For this reason, critically ill patients are at risk of developing micronutrient deficiency. Unfortunately, traditional markers used to assess micronutrient status in healthy patients are often unreliable in the critically ill [2]. Assessment of micronutrients in this patient population is challenging due to multiple factors that include illness severity, nutritional status, medication use, and undesirable consequences of medical and surgical interventions. Determining micronutrient status in infants and children is further complicated given their immature organ function and specific metabolic demands that differ significantly from that of the adult population. This review aims to systematically present the most current knowledge on evaluating micronutrient status in critically ill children, and identify opportunities and challenges that can guide future research in this area.

## 2. Water Soluble Vitamins

### 2.1. B Vitamins

#### 2.1.1. Introduction

The B vitamins are composed of 8 essential water-soluble vitamins that function as co-factors for various metabolic processes (Table 1). Folate, cobalamin, and thiamine have drawn great interest due to the detrimental clinical manifestations that result from deficiency states. Folate is particularly important in fetal development as deficiency may result in life threatening neural tube defects [3]. Cobalamin deficiency during this stage can also lead to neurologic sequelae and poor development [4,5]. Thiamine is essential for various cellular processes and deficiency may result in Wernicke–Korsakoff syndrome, beriberi, and death [6,7,8,9,10,11]. In general, deficiency of the B vitamins in children may impair growth and development due to their essential cellular and biochemical functions [3,12]. 

#### 2.1.2. Assessment

Due to their multiple roles in regulating various metabolic reactions, indirect assessment of vitamin B status can be achieved by functional assays that measure their activity as related to a specific enzyme or metabolite (Table 2). More recently, the availability of high performance liquid chromatography (HPLC) has allowed for direct measurements of these vitamin complexes in blood and other organs [3,5,6,8,11,13,14,15]. However, plasma concentrations of riboflavin, flavin mononucleotide, and flavin adenine dinucleotide have been shown to be significantly affected by acute inflammation, and show transient decreases that might not reflect true body storage. For this reason, some have advocated for the use of erythrocyte concentrations as a better indicator of their status, especially during times of acute illness and inflammation [11,16,17].

#### 2.1.3. Vitamin B in Critical Illness

Assessment of water soluble vitamins can be particularly challenging in pediatric patients given the present lack of understanding of micronutrient status in the critically ill. At baseline, children have varying levels of B vitamins due to normal growth and development [9,18,19,20]. Critically ill children are even more prone to deficiency and abnormal concentrations due to a hypermetabolic state, decreased intestinal absorption, insufficient intake, excretion, drug-related fluctuations, and underlying metabolic diseases [21]. Additionally, due to the complex cellular and biochemical mechanisms involving B vitamins and the imbalances that occur during critical illness, functional assays for enzyme activity and indirect measurements of biomarkers for these vitamins may not be as accurate as direct quantification of the active B vitamins. Given these challenges, there is limited data on the assessment of vitamin B status in the critically ill pediatric population. 

In adults, low plasma thiamine levels are common and are associated with increased mortality in patients in intensive care units (ICU) [6]. In one study, up to 12.5% of pediatric intensive care unit (PICU) patients were found to be thiamine deficient based on erythrocyte transketolase, while pyridoxine deficiency was not observed and riboflavin deficiency occurred at a much lower rate [10]. A similar trend was also seen in children receiving chemotherapy, suggesting that thiamine deficiency is an unrecognized problem in many children with critical illness. The authors also noted that due to the lack of specific signs and symptoms, screening for clinical manifestations of Wernicke encephalopathy was insufficient for the diagnosis of thiamine deficiency. Other studies in adults do show decreased plasma levels of pyridoxal 5′-phosphate in different inflammatory conditions, mostly due to alterations in its tissue distribution and increased catabolism [22,23]. The pyridoxic acid to pyridoxal plus pyridoxal 5′-phosphate ratio (PA:(PL + PLP)) has been shown to be a useful marker of vitamin B_6_ catabolism during states of inflammation [23]. Similarly, vitamin B_6_ increases with systemic inflammation, as measured by erythrocyte pyridoxal phosphate levels [11,24]. Another study showed that plasma concentrations of thiamine were decreased in association with systemic inflammation based on C-reactive protein (CRP) levels [9]. However, no association between low thiamine levels and outcome variables such as mortality, length of stay, or ventilator dependence was observed. Higher vitamin B_12_ levels were found in critically ill adults and showed a correlation with CRP levels and higher mortality [25,26]. However, assessment of these vitamins in the pediatric population has not yet been studied.

### 2.2. Vitamin C

#### 2.2.1. Introduction

Vitamin C (ascorbic acid) is an essential vitamin that functions physiologically as a water-soluble antioxidant. It is found in both the intra- and extracellular compartments in blood where it reduces harmful oxidants, improves immune function, plays a major role in wound healing, facilitates uptake of non-heme iron, and acts as a co-factor for many enzymes [27,28,29,30]. Vitamin C is also essential for scavenging reactive oxygen species (ROS) which may damage lipids, proteins, and deoxyribonucleic acid (DNA) [27,31]. Toxicity is rare, as excess is excreted in the urine [32]. However, large doses may cause gastrointestinal discomfort [32]. Deficiency may result in poor wound healing, lethargy, or symptoms of scurvy such as perifollicular petechiae, gingivitis, and bleeding from mucus membranes [30]. Infants may develop signs of inadequate intake of vitamin C particularly in the first year of life if they are fed unsupplemented cow’s milk or if they suffer from gastrointestinal malabsorption [33,34]. Children may exhibit similar signs of deficiency in addition to poor formation of bone osteoid, leg swelling, subperiosteal hemorrhage, hemarthrosis, and iron deficiency [33]. 

#### 2.2.2. Assessment

Methods for measuring vitamin C include several enzymatic, spectrophotometric, and chromatographic assays. HPLC is an accurate and efficient method for analysis [30,35]. However, it is expensive and not routinely available. In healthy adults, the average level of vitamin C is 20 mg/kg by body weight, and the mean plasma concentration is 45–85 μmol/L [29,32,35,36]. Significantly higher concentrations are found in immune cells [30]. However, there is relatively little long-term body storage of vitamin C and thus, concentrations are largely dependent on daily intake. Deficiency is defined as serum concentrations of less than 20 μmol/L [29]. There is no standard for serum or urine vitamin C concentrations in the pediatric population. Similarly, there are no set recommendations for dietary intake for normal healthy infants. There is active placental transfer in utero and thus, newborn levels most closely reflect those of maternal plasma concentrations [33]. Vitamin C is also present in breast milk in higher concentrations than in cow’s milk [33]. 

#### 2.2.3. Vitamin C in Critical Illness

Interpreting serum levels in the acutely ill is difficult due to variations in absorption, distribution, consumption, and renal excretion [28,29]. Due to the unstable nature of the compound and the lack of a gold standard technique for measuring levels, serum analysis for vitamin C continues to be a challenge in most clinical settings. Many groups have studied the association between vitamin C and oxidative stress resulting from sepsis, ischemia/reperfusion injury, hemorrhage, multiple organ failure, and post-cardiac surgery [28,30,32]. As a biomarker, vitamin C decreases in states of oxidative stress, likely related to increased immune cell turnover and changes in body compartment distribution [28,29]. There is limited data regarding appropriate vitamin C levels in pediatric patients. However, as in adults, critically ill children may require higher doses of vitamin C supplementation as it supports microcirculatory changes and immune function [28,30]. Despite being an important factor in the body’s defense against oxidative stress, the difficulty in measuring vitamin C concentrations and interpreting their changes in critically ill children has resulted in a paucity of data. Further research aimed at understanding the role of vitamin C levels in the pediatric population is required in order to assess the clinical utility of measuring concentrations. 

## 3. Fat Soluble Vitamins

### 3.1. Vitamin A

#### 3.1.1. Introduction

Vitamin A was the first vitamin to be discovered. It exists in three forms: retinal, retinoic acid, and retinol, with the latter being routinely used to measure its status [37]. Given its fat-soluble nature, vitamin A circulates in plasma bound to a water-soluble complex of retinol-binding protein (RBP) and transthyretin. It is stored in the liver, adipose tissue, and the adrenal glands [38]. Dietary sources of vitamin A include animal products which are rich in preformed vitamin A (retinyl esters), fruits and vegetables high in carotenoids, and the provitamin form of vitamin A [39]. Although its main role pertains to visual health, vitamin A serves other functions in the body that allow for adequate growth, immune and reproductive function, and epithelial integrity [37,40]. 

#### 3.1.2. Assessment

Measurement of retinol concentration in serum is the main test to assess vitamin A status in the clinical setting. Adequate plasma retinol levels in older children and adults fall between 0.7–2.8 µmol/L. However, special considerations must be taken into account when assessing vitamin A status in preterm infants [41]. Preterm neonates are born with low hepatic reserves of vitamin A and significantly lower concentrations of plasma retinol and RBP, with the latter being a result of both liver immaturity and interruption of transplacental transfer of RBP to the fetus [42]. Other assessment methods have thus been proposed in this setting, including the plasma retinol/RBP molar ratio, and the relative dose response of retinol (RDR), which measures changes in RBP levels following administration of a single dose of vitamin A [43,44] and takes into account the mobilization from hepatic stores during states of deficiency. The use of these methods has been particularly useful in assessing vitamin A status in preterm neonates given the association of its deficiency with the development of bronchopulmonary dysplasia (BPD) and retinopathy of prematurity (ROP). Apart from serum biomarkers, other markers of vitamin A status include those that assess visual function, including measurement of dark adaptation, electroretinography, and pupillary threshold testing [45]. As for vitamin A toxicity, measurement of fasting plasma retinyl esters is the test of choice, although some conditions such as liver disease and malnutrition can raise its levels despite normal vitamin A status [45]. Caution must therefore be taken when interpreting levels of retinyl esters in those with pre-existing conditions and in the critically ill.

#### 3.1.3. Vitamin A in Critical Illness

Interpretation of retinol levels in the critically ill must also take into account the fact that serum RBP levels decrease significantly as other acute phase reactants are synthesized by the liver in times of physiologic stress. The elevation in levels of CRP that occurs in the setting of acute infections and other inflammatory conditions has shown a good correlation with dropping levels of serum retinol, which is likely the result of decreased hepatic RBP synthesis [46]. Also, the vasodilation and resulting leakage may lead to the extravasation of RBP and other lipids into the extracellular compartment [47]. The drop in circulating lipid levels that occurs during the inflammatory response compromises the interpretation of fat-soluble vitamin status in the critically ill. The transient drop that is seen in circulating levels of vitamin A (and other fat-soluble vitamins) in the setting of inflammation disappears once corrected for lipid levels [48,49]. The duration of an inflammatory state also impairs vitamin A status and increases the risk of deficiency in this patient population. Critically ill children are at particular risk for developing vitamin A deficiency due to inadequate nutritional provision either through the parenteral (e.g., light-induced vitamin degradation, adsorption via infusion lines) or enteral routes (e.g., malabsorption syndromes, bowel immaturity) [40]. Those born to vitamin A-deficient mothers are also at risk [45]. Additionally, urinary excretion of retinol increases significantly in the setting of infection, and is even more pronounced in sepsis [50]. Stephensen et al. found that almost a third of patients with severe infections excreted amounts of retinol in urine equivalent to 50% of the recommended dietary allowance [51]. Similarly, Mitra et al. estimated the effects of a single episode of sepsis on the vitamin A status of an average 2-year-old child. In developed countries, the urinary loss of retinol would translate into depletion of nearly 20% of the total liver stores. This number rises to almost 75% in developing countries, where children have significantly less hepatic reserves of vitamin A [50].

Although it is well known that vitamin A deficiency is associated with respiratory complications in the critically ill preterm neonate, data supporting its supplementation for their prevention is mixed [41]. A recent systematic review by Darlow et al. evaluated the results from 11 trials and found a small benefit of vitamin A supplementation in reducing the risk of mortality and the development of chronic lung disease in very low birth weight (VLBW) infants [40]. Another systematic review by the same author addressed the effectiveness of supplementation on ROP and found a positive trend towards a beneficial effect, although it did not reach statistical significance [52]. In older children, the effects of vitamin A supplementation proved to be of no benefit in the treatment of severe lower respiratory tract infections [53]. However, this was not the case for children with severe measles, in which provision of vitamin A proved to be beneficial in reducing the mortality rate and other associated complications [54]. 

### 3.2. Vitamin D

#### 3.2.1. Introduction

The nutritional forms of this fat-soluble vitamin are ergocalcipherol (vitamin D_2_) and cholecalciferol (vitamin D_3_). The former is derived from plant sterols, whereas the latter is synthesized in the skin from its precursor, 7-dehydrocholesterol, under the catalysis of ultraviolet radiation exposure [55]. Vitamin D has many different metabolites, with 1,25-dihydroxyvitamin D (1,25(OH)_2_D) being the most physiologically active form [56,57]. A sequence of hydroxylation reactions, first in the liver and then in the kidney, results in the activation of vitamin D into 1,25(OH)_2_D. Hydroxylation of vitamin D in the liver leads to the formation of its main circulating, yet inactive form, 25-hydroxyvitamin D (25(OH)D). The physiologically active 1,25(OH)_2_D, in conjunction with parathyroid hormone (PTH), calcitonin, and sex steroids, acts mainly on bone and the small intestine to regulate calcium homeostasis. Vitamin D metabolism is tightly regulated by serum levels of calcium, phosphorus, PTH, and 1,25(OH)_2_D itself [55]. 

Risk factors associated to vitamin D deficiency can be environmental (e.g., limited exposure to sunlight and/or ultraviolet radiation) or inherent to the host (e.g., dark pigmented skin, limited dietary intake, malabsorptive syndromes, chronic illnesses) [58]. Vitamin D deficiency in children can result in decreased bone mineralization and rickets, which can lead to stunted growth development and an increased risk of fractures. Also, low levels of vitamin D may impair the innate and adaptive immune responses, and increase the risk of developing other chronic diseases such as type 1 diabetes and other autoimmune disorders [58,59,60].

#### 3.2.2. Assessment

Given its lipophilic nature as a steroid hormone, vitamin D is transported in plasma almost in its entirety by a liver-derived glycoprotein known as vitamin D-binding protein (VDBP) [61]. Although VDBP is the major carrier of vitamin D in the bloodstream, it does not ultimately affect the levels of free or bioavailable vitamin D, and its absence does not seem to affect the actions of vitamin D in target tissues [62,63]. For this reason, calculation of the bioavailable 25(OH)D—25(OH)D not bound to VDBP—has been proposed more recently as a more reliable marker of vitamin D status [64,65]. Serum 25(OH)D has a half-life of 14–20 days and accurately correlates with the amount of vitamin D present in body stores. The Institute of Medicine has defined levels of cord serum 25(OH)D at which there is an increased risk of vitamin D deficiency (<30 nmol/L), an adequate threshold (40 nmol/L), and sufficiency (≥50 nmol/L) [66]. 

Whereas vitamin D is required to maintain normal calcium levels in adults, calcium homeostasis and bone development are not dependent on vitamin D levels during the fetal period [67]. Several mechanisms of maternal adaptation for calcium provision alongside fetal parathyroid hormone-related protein (PTHrP) ensure that calcium levels remains optimal to promote the adequate development of the fetal skeleton [68]. Since 1,25(OH)_2_D does not cross the placenta, its blood levels in the fetus are very low. Following birth, the quick drop in calcium levels leads to a rise in blood levels of PTH and subsequently 1,25(OH)_2_D, the latter reaching adult levels approximately two days after delivery [68,69]. In that sense, although the fetus seems to be unaffected by vitamin D levels during pregnancy, there is an increased risk of hypocalcemia during the neonatal period of those born to vitamin D-deficient mothers. Although the data is mixed, vitamin D supplementation of pregnant mothers seems to allow neonates to better withstand the changes in calcium metabolism that occur postnatally, and the bone accretion rates seen during this period [58,66].

#### 3.2.3. Vitamin D in Critical Illness

The physiologic and metabolic stress that is seen in the critically ill can lead to acute changes in vitamin and mineral concentrations. Critically ill patients can be particularly affected by vitamin D deficiency, given the resulting detrimental effects on calcium homeostasis, immune function, and the oxidant-antioxidant balance [70,71]. Similar to what occurs with other micronutrients, the duration of inflammation may be an important factor in determining whether a low concentration of vitamin D is truly a marker of deficiency. Reid et al. described a rapid and significant drop of approximately 40% in plasma levels of 25(OH)D in adult patients during the evolution of the systemic inflammatory response in the immediate postoperative period [72]. Overall, the prevalence of vitamin D levels <50 nmol/L in patients admitted to PICU ranges from 28–69% [70,73,74,75,76,77,78,79]. A multicenter study across Canada found that almost 70% of patients had levels of 25(OH)D <50 nmol/L on admission [70]. Consistent with what is known from studies performed in the adult population, this study also showed an association between low admission vitamin D levels and both increased illness severity scores and longer PICU length of stay. Similarly, single-institution studies in the United States and India separately found that nearly 40% of patients admitted to the PICU had 25(OH)D levels below 50 nmol/L, more commonly during the winter months and in those with darker skin [73,77]. There is also an inverse relationship between 25(OH)D levels and illness severity scores at admission, need for vasopressor support during ICU stay, and mean duration of mechanical ventilation. Another study in a pediatric burn unit showed that more than half of all patients presenting with severe burns (>25% total body surface area) had levels of 25(OH)D below 37.3 nmol/L, with a relationship observed between hypovitaminosis and the presence of inhalational injury, and a trend showing an association between low vitamin D and burn severity [80]. These findings are likely the result of a combination of factors that include electrolyte derangements (i.e., hypocalcemia, hypomagnesemia, and hypophosphatemia), disruption of the epidermis and vitamin D synthesis in the skin, limited sun exposure, and prolonged immobilization among others [80]. 

Levels of 25(OH)D in the critically ill should be interpreted with caution, as dysfunction of other organs (i.e., parathyroid, kidney) may limit the conversion of 25(OH)D to 1,25(OH)_2_D [70]. Also, the acute fluid loading that is normally seen in this setting can cause a hemodilution effect and lower the levels of both 25(OH)D and 1,25(OH)_2_D [81]. Data from a large cohort, single-institution study highlighted the strong association between plasma 25(OH)D and both CRP and albumin levels [82]. These findings support the unreliability of interpreting levels of plasma 25(OH)D in critically ill patients. A single-institution study by Madden et al. showed that critically ill children had lower levels of VDBP compared to the general pediatric population [65]. An acute phase reactant, decreasing VDBP in turn increases the level of bioavailable 25(OH)D and thus may serve as a protective mechanism against the detrimental effects of hypovitaminosis D in the critical care setting. It is clear that vitamin D insufficiency or deficiency is highly prevalent in the PICU population and is associated with worse outcomes in this setting. Providers should be aware of the different risk factors for deficiency that may make some patients more susceptible than others, and implement adequate strategies for supplementation.

### 3.3. Vitamin E

#### 3.3.1. Introduction

Vitamin E is a lipid-soluble antioxidant with 8 natural isoforms [83,84]. Vitamin E plays a major role in the balance between the body’s natural antioxidant system and oxidative damage from ROS such as superoxide anions, hydrogen peroxide, and hydroxyl radicals [83,84]. Leaving unchecked, these compounds may result in protein oxidation, lipid peroxidation, and DNA damage leading to direct tissue injury [85,86,87]. Vitamin E is an important free radical scavenger that protects cells from oxidative damage [83,85,87]. 

#### 3.3.2. Assessment

Alpha-tocopherol is the primary determinant for vitamin E status in the body, as it is the active isoform [83]. Alpha-tocopherol is also the most abundant form given the presence of a hepatic transfer protein that prevents it from being excreted in bile with the other isoforms of vitamin E [88]. It is mostly found in cell membranes, and normal healthy adults have a serum concentration of >11.5 μmol/L [87]. There is, however, no established reference range for children. There have been different approaches to measuring α-tocopherol concentrations. The most common method for measuring α-tocopherol levels is based on plasma concentrations using HPLC [85,86,87,89,90]. Several groups suggest measuring ratios between α-tocopherol and serum lipid concentrations due to the relationship of plasma lipid levels and the lipid-soluble nature of vitamin E [87,90,91]. Though plasma levels of α-tocopherol are strongly associated with lipid concentrations, there is not enough data to establish a standard method or reference range for these measurements [87,90]. 

#### 3.3.3. Vitamin E in Critical Illness

Oxidative stress is associated with critical illness and its related complications of multiple organ failure, sepsis, and mortality [30,83,92]. This is likely due to a combination of factors including ischemia-reperfusion injury, systemic inflammatory response, fluid shifts in body compartments, and a host of other physiologic responses to injury or disease. Many neonatal diseases and complications, such as respiratory distress syndrome, necrotizing enterocolitis (NEC), chronic lung disease, ROP, and intraventricular hemorrhage are related to increased oxidative stress as well [93]. However, there are mixed reports of vitamin E status in critical illness. In one study, up to 21% of adult surgical ICU patients were found to have low plasma concentrations of α-tocopherol [85,87], while another group found that vitamin E status did not correlate with critical illness [25]. However, interpretation of vitamin E levels in this setting should take into consideration the redistribution of cholesterol that occurs as part of the systemic inflammatory response. Conway et al. evaluated the effect of inflammation on plasma α-tocopherol levels in adult patients undergoing hip arthroplasty [49]. Although there was a significant transient reduction in α-tocopherol levels during the postoperative period, this change disappeared once adjusted for cholesterol levels. These findings warn against measuring isolated vitamin E levels in the critically ill and suggest adjusting its ratio to cholesterol (or total lipids) for a more reliable assessment of vitamin E status. Studies evaluating vitamin E status in PICU patients are currently lacking, and further investigation is required in order to understand its specific roles in modulating oxidative damages during critical illness.

### 3.4. Vitamin K

#### 3.4.1. Introduction

Vitamin K refers to a group of fat-soluble vitamins that include phylloquinone (vitamin K1) and a collection of structurally similar vitamers called menaquinones (vitamin K2) [94]. Phylloquinone constitutes the majority of vitamin K found in diet and is present in high amounts in green leafy vegetables such as kale, spinach, and broccoli [95]. Menaquinones, found in eggs, meat, and fermented products, can also be synthesized by intestinal bacteria and various organs in the body [96,97,98,99]. Vitamin K serves as a co-factor for γ-glutamyl carboxylase, which participates in post-translational modification and activation of proteins that are essential to coagulation, calcium homeostasis, and vascular health [94]. Among these are clotting factors II, VII, IX, and X, and anti-coagulant proteins C and S of the coagulation cascade. 

While the major form of vitamin K storage in adults is menaquinone, phylloquinone makes up most of the hepatic content of vitamin K in neonates and breast milk [100]. Due to the neonate’s poor vitamin K reserves, as well as the low content of vitamin K in breastmilk, both term and preterm infants are at risk for vitamin K deficiency and, more seriously, vitamin K deficiency bleeding (VKDB)—a condition characterized by spontaneous bleeding due to low levels of clotting factors [101,102]. However, with adequate prophylaxis and formula supplementation, VKDB should be preventable and is now rare in developed countries. 

#### 3.4.2. Assessment

Traditional methods of vitamin K assessment include evaluation of coagulation functions such as prothrombin time (PT) and international normalized ratio (INR). However, a prolonged PT is not specific for vitamin K deficiency and is only seen with a large drop in factor II levels [98]. Furthermore, coagulation function is an especially poor indicator of vitamin K status in infants [103,104]. Neonatal levels of clotting factors are less than 70% of those in adults and there is a poor correlation between vitamin K prophylaxis and meaningful changes in coagulation functions. 

Direct measurement of plasma phylloquinone is another method to assess vitamin K status. Plasma phylloquinone, which makes up the majority of circulating vitamin K, has been shown to correlate well with dietary intake [105,106] and serves as a good indicator of overall vitamin K status in adults [107]. However, plasma phylloquinone in the normal population varies across a wide range [108]. Additionally, since the majority of phylloquinone in circulation is associated with triglyceride-rich, very low density lipoprotein (VLDL), assessment of plasma phylloquinone needs to take into account triglyceride levels [108]. In neonates, plasma phylloquinone is extremely low at birth but shows a marked increase with oral or intramuscular administration of vitamin K [109]. However, even with the depot effect from intramuscular injection of vitamin K, the rise in phylloquinone levels is not sustainable, as exclusively breastfed infants at six months of age still show significantly lower plasma phylloquinone concentrations compared to those fed with fortified formula [110]. 

Another commonly used marker of vitamin K status is protein-induced vitamin K absence-II (PIVKA-II), a mixture of functionally abnormal and undercarboxylated molecules of prothrombin that only become detectable in the serum in states of vitamin K deficiency. PIVKA-II is a sensitive indicator of vitamin K since its levels begin to rise even before any abnormality in PT is detected [102]. With a long half-life of 60 h, PIVKA-II measurement also allows for diagnosing vitamin K deficiency even after corrective therapies have been initiated [102]. However, the utility of PIVKA-II in infants is more debatable. Elevated PIVKA-II is found at birth in almost half of term infants and, given the immature coagulation function in the neonates, the significance of this finding remains unclear [111]. PIVKA-II is commonly measured in many studies that evaluate the efficacy of continuing vitamin K supplementation for exclusively breast-fed infants. Compared to those receiving intramuscular prophylaxis at birth and those who are bottle fed, breastfed infants have significantly higher PIVKA-II levels at 3 months of age [112]. A single dose of either oral or intramuscular injection of vitamin K at birth is insufficient to suppress PIVKA-II at 3 months of age [113] and additional oral supplementation beyond the postnatal period is needed in order to prevent biochemical evidence of vitamin K deficiency [114,115]. However, the current North American guideline for vitamin K prophylaxis in healthy term infants remains a single dose of 1 mg of phylloquinone by intramuscular injection at birth [116]. 

#### 3.4.3. Vitamin K in Critical Illness

In the face of acute inflammation, lipid soluble antioxidants such as vitamin A and E are known to transiently decrease in part due to a fall in cholesterol and triglyceride concentrations [48,49]. Vitamin K, due to its association with VLDL, shows a linear correlation with plasma triglyceride levels and thus displays a similar pattern in response to systemic inflammation [108]. Plasma phylloquinone concentrations therefore should be used with caution when determining the vitamin K status of patients with critical illness. An important factor that could affect the vitamin K status in critically ill children is the prolonged use of antibiotics. Prolonged PT, or hypoprothrombinemia, has been associated with the use of certain antibiotics, especially those containing the *N*-methyl-thiotetrazole (NMTT) side chain, such as third-generation cephalosporins [117]. Proposed mechanisms include their direct inhibitory effect on the γ-carboxylation of vitamin K-dependent clotting factors and disruption of the intestinal flora that accounts for menaquinone production. Regardless of the true mechanism, the effect of antibiotics on vitamin K status in critically ill children is most evident in those with protein energy malnutrition or those on a prolonged course (10 days or more) [118,119]. However, prophylactic administration of vitamin K in this population does not seem to prevent the development of hypoprothrombinemia [119]. 

Vitamin K prophylaxis has been extensively studied in preterm infants in order to prevent the devastating complications of VKDB [120,121,122,123,124,125]. Multiple doses and routes of administration have been investigated, and all of them consistently show a dramatic increase in plasma phylloquinone levels, possibly due in part to the immature hepatic metabolism of vitamin K in premature infants. PIVKA-II levels are also low-to-undetectable following prophylaxis, indicating adequate provision of vitamin K with any route of administration. Compared to intramuscular injection, intravenous prophylaxis is more likely to produce vitamin K overload in preterm neonates, yet it is less effective in sustaining the plasma phylloquinone levels [123]. However, for preterm infants whose size precludes intramuscular injection and in whom enteral feeding is yet to be established, parenteral administration of vitamin K remains a feasible option for supplementation [121]. 

## 4. Trace Elements

### 4.1. Iron

#### 4.1.1. Introduction

Iron is an essential element that plays many central, but sometimes conflicting, roles in the body. It is critical for oxygen transportation and energy generation as a part of the heme molecule and many metabolic enzymes [38]. On the other hand, its roles in ROS formation through the Fenton reaction have been theorized as a contributing etiology for a number of detrimental transfusion-associated neonatal diseases, such as ROP and NEC [126,127]. Adequate iron status is vital for normal neurological development, as iron deficiency results in defects in myelinogenesis, neurotransmission, and metabolic activity of the neonatal brain [128]. Additionally, dysregulation of iron storage has been implicated in hypoxic ischemic encephalopathy and the associated periventricular white matter damage [129,130]. Assessment of iron status in preterm infants and iron supplementation during the first few months of life in this population have become a cornerstone of neonatal care [131]. 

Significant differences exist between neonatal iron metabolism and that of older children or adults. Preterm infants are born with significantly less total body iron content compared to term infants, mostly due to a shorter period of iron accumulation from the mother [132]. Newborns display high levels of ferritin due to increased iron loads from continuing erythrocyte degradation in the face of decreased erythropoiesis [133,134]. While iron metabolism is tightly regulated in adults, iron levels in premature infants are much more susceptible to environmental influences such as dietary supplements, phlebotomy, and transfusion. Infants lack the ability to control iron absorption from the gastrointestinal tract and their iron levels vary in accordance with dietary iron supplementation [132]. Erythrocyte transfusion in premature infants often results in iron overload [132,135] while phlebotomizing is a major contributor to iron-deficiency anemia in neonates, as each gram of hemoglobin removed results in a loss of 3.4 mg of iron [132]. 

#### 4.1.2. Assessment

In adults, total iron content approximates 35–45 mg/kg, with almost two thirds being incorporated into erythrocytes [136]. As iron losses through blood, sweat, skin, and intestinal cell sloughing are quite minimal and its absorption from the gastrointestinal tract is a highly regulated process, the body relies heavily on turnover from erythrocytes to maintain adequate iron stores. Iron is mostly stored in the liver and tissue macrophages in the form of intracellular ferritin molecules. In blood, it is transported from the sites of storage to sites of utilization (e.g., bone marrow and muscle) via transferrin. However, measuring the plasma concentration of iron does not accurately reflect the total body storage, as only 1/1000 of the total iron content exists in circulating plasma [136]. 

The zinc protoporphyrin/heme (ZnPP/H) ratio has been regarded as a more sensitive marker for iron deficiency, although it is not as commonly used as ferritin or transferrin [137]. Since protoporphyrin IX in erythrocytes incorporates zinc in place of iron during times of limited iron availability, an elevated ratio indicates functional iron deficiency, and has been shown to correlate well with other markers of iron status [138]. In neonates, ZnPP/H is found to be inversely proportional with gestational age, indicating more severe iron deficiency in more premature infants [139]. The ZnPP/H ratio also responds accordingly to changes in the body iron content as it decreases with iron supplementation and blood transfusion but increases with recombinant erythropoietin treatment and erythropoiesis [139,140]. However, ZnPP/H also varies with age and only measures functional iron status. Therefore, it is best used when trended over time and in conjunction with other iron biomarkers, as well as hematocrit and reticulocyte count. 

#### 4.1.3. Iron in Critical Illness

The body responds to serious infection or critical illness by lowering its iron availability. This is thought to be a defense mechanism against invading organisms by limiting their iron utilization [141,142]. The “master regulator of iron metabolism”, hepcidin, is upregulated in times of inflammation and sequesters iron in its ferritin form by inhibiting iron export both in macrophages (sequestering iron) and in enterocytes (preventing the absorption of dietary iron) [143]. This sequestration of iron is accountable for the condition known as anemia of inflammation, which is also associated with a rise in ferritin and decreased iron, transferrin, and transferrin saturation. 

Evaluating iron status in critically ill children is a challenge due to a combination of factors, such as varied transfusion protocols, changing iron storage during development, different iron supplementation regimens and degrees of absorption, as well as the confounding effects of inflammation. In a study of 46 patients admitted to the neonatal intensive care unit (NICU), extremely critical infants—defined as those with 10 points or more on the score for neonatal acute physiology (SNAP)—had significantly lower serum iron levels than those with a lower SNAP [144]. A similar study in children in the PICU found an inverse correlation between serum iron levels and the pediatric risk of mortality (PRISM) score, reinforcing the concept of iron sequestration in critical illness [145]. However, no other indices of iron status were assessed in either of these studies. 

Although an in-depth discussion on the varying practices of transfusion in children is beyond the scope of this review, it is well-known that anemia and the associated risks of transfusion continue to be major problems faced by children in the ICU [146]. In a prospective observational study of 30 PICUs, 74% of patients were found to be anemic and almost half received transfusions with a wide variation in transfusion-trigger hemoglobin levels [147]. There is an association between transfusion and increased mortality, as well as prolonged ventilator dependence and ICU stay. Additionally, phlebotomy is the major contributor to daily blood loss, and aggressive phlebotomy significantly increases the incidence of transfusion events. Transfusion is also known to significantly increase the levels of non-transferrin bound iron (NTBI), especially in preterm infants, and thus theoretically contributes to increased oxidative injuries in this vulnerable population [148,149,150]. However, this increase in NTBI is transient and a clear causative relationship between NTBI and actual ROS-induced organ injuries has not been established. 

### 4.2. Zinc

#### 4.2.1. Introduction

Zinc is an essential element that serves a wide range of bodily functions such as catalyzing metabolic reactions, providing structural support for important proteins, and regulating gene expression [151]. The symptoms of zinc deficiency are diverse and have been well documented due to its many functions in maintaining the body’s homeostasis. Mild to moderate zinc deficiency can result in poor appetite, lethargy, recurrent infections, or growth retardation while severe deficiency manifests as bullous dermatitis, alopecia, diarrhea, hypogonadism in males, neurosensory impairment, depressed immune function, and poor wound healing [152]. The body has developed a remarkable ability to conserve zinc during times of low dietary intake, perhaps due to its vital roles in overall metabolism. Urinary and gastrointestinal excretion of zinc significantly reduces in healthy adults placed on a low-zinc diet, resulting in a loss of less than 5% of the body’s total zinc content [153,154].

#### 4.2.2. Assessment

Despite being a central component of the ubiquitous zinc-finger domain-containing proteins [155], there is a very small pool of exchangeable zinc that can respond quickly to both dietary restriction or supplementation [151]. Serum zinc is a component of this small, exchangeable pool and therefore is very sensitive to changes in dietary intake. In the bloodstream, zinc is transported with albumin (70%) and α_2_-macroglobulin (18%), among other proteins [156]. Plasma zinc declines sharply with severe restriction, increases with zinc supplementation, and promptly returns to baseline after cessation of supplementation [157,158]. Plasma zinc is a good indicator of zinc intake in the absence of systemic inflammation. However, it does not reflect the total zinc content, which mostly participates in metabolic activities, and is not readily available for exchange with the environment. 

Transportation of zinc across the plasma membrane depends on two major classes of transporters that work in opposite directions. The Zrt-, Irt-like Protein (ZIP) family of transporters shift zinc from the cell’s exterior to the cytoplasm while the Zn transporter (ZnT) family move zinc out of the cytoplasm into other cellular organelles or the extracellular environment [159]. In the intracellular compartment, zinc is temporarily stored in metallothionein proteins, which exist in four different isoforms and are present throughout the body, especially in the liver, kidney, intestine, and pancreas [160,161]. These proteins are also likely to participate in the regulation of plasma zinc levels in accordance with dietary intake. The levels of intestinal metallothionein rise with increased zinc intake, resulting in reduced uptake into the bloodstream, while decreased pancreatic and renal metallothioneins seen with dietary deficiency may help to limit fecal and urinary losses [161]. Alkaline phosphatase, one of the earliest zinc-containing enzymes to be identified, also shows sensitivity to dietary intake and has sometimes been used as a surrogate marker for zinc status [162]. 

#### 4.2.3. Zinc in Critical Illness

Acute inflammation induces redistribution of zinc and is responsible for a shift from blood to the liver, where zinc is utilized for hepatic production of acute phase reactants [163,164]. This results in the depressed serum zinc levels that accompany conditions such as cancer, infection, post-bone marrow transplant, or endotoxin challenge [151,165]. In addition to systemic inflammation, failure to meet the recommendations for dietary intake also contributes to low serum zinc levels seen in PICU patients [21]. Microarray analyses revealed the dysregulation of many genes involved in zinc homeostasis in children with septic shock [166]. Non-survivors of septic shock display significantly higher levels of metallothionein and correspondingly lower levels of serum zinc compared to survivors. Plasma zinc levels showed an inverse correlation with CRP and interleukin (IL)-6, as well as with the degree of organ failure in another study of critically ill children [167]. Analyses of baseline zinc levels revealed hypozincemia in more than 80% of the children enrolled in the multicenter Critical Illness Stress Induced Immune Suppression (CRISIS) Prevention Trial [168]. There was also a correlation between low serum zinc levels and lymphopenia, defined as lymphocyte count <1000 cells/mm^3^. This correlation between zinc levels and lymphocyte count was confirmed in another study of critically ill Brazilian children, where more than a third of the patients also showed evidence of malnutrition [169]. 

Plasma zinc levels in VLBW premature infants display a marked decrease during the first 8 weeks of life, a trend parallel to term infants [170,171]. This most likely is a result of increased metabolic demands coupled with low tissue reserves, and is evident by the development of symptoms of zinc deficiency in parenteral nutrition (PN)-dependent premature infants despite being supplemented with 146% to 195% of the recommended daily zinc intake [172]. Severe deficiency, with zinc levels <7.65 μmol/L, is still discovered in 6.6% of VLBW infants meeting the European Society for Paediatric Gastroenterology Hepatology and Nutrition (ESPGHAN) guidelines for zinc intake [173]. Low birthweight and history of NEC have been associated with an increased risk of developing zinc deficiency in this vulnerable population. 

Zinc supplementation has been extensively studied with the goal of improving immune modulation and outcomes, especially in serious infection. Although serum zinc levels consistently increase with various forms of supplementation, these studies and trials have been met with mixed results. In one of the largest of these trials to date, supplementation with a mixture of zinc, selenium, glutamine, and metoclopramide (CRISIS prevention trial) showed no reduction in the rate of nosocomial infections in immunocompetent patients but a possible effect in the small number of immunocompromised children [174]. Intravenous zinc supplementation in PICU patients is feasible and increases plasma zinc levels but shows no effect on lymphocyte count [175]. Oral zinc supplementation has been attempted in many studies in developing countries with the goal of shortening the duration of severe pneumonia. Although zinc supplementation at the dose of 20 mg/day may be beneficial in cases of very severe pneumonia [176], the current evidence does not support this therapy as an effective adjunct treatment for pediatric lower respiratory tract infections [177,178,179,180,181]. 

### 4.3. Selenium

#### 4.3.1. Introduction

Selenium was initially identified as a critical factor in the pathogenesis of Keshan disease, a form of juvenile cardiomyopathy. It is now recognized to play a key role in regulating immune, reproductive, neurological, cardiovascular, and endocrine functions [182]. Its main function is to serve as the co-factor for enzymes involved in protection against oxidative damage, most notably glutathione peroxidase (GSHpx) [182]. Assessment of selenium functional status therefore usually involves the measurement of GSHpx activity. Diet provides the most important source of selenium in humans. Its content in soil significantly impacts the levels of selenium intake across different geographic regions in the world. Low soil levels of selenium are found in South America, parts of Europe, and a large area across mainland China, where diseases associated with selenium deficiency were first characterized [183]. 

#### 4.3.2. Assessment

Selenium in blood is mostly associated with selenoprotein P (40–70%), GSHpx (20–40%), and albumin (6–10%) [182]. Serum levels reflect short-term storage and represent less than 1% of the total body selenium content. However, it is the most commonly used parameter to assess selenium status [184]. Erythrocyte, hair, and nail selenium levels might be more useful indicators of long-term status, despite certain limitations [183]. GSHpx activity has been commonly measured as an adjunct assessment of selenium functional status. Studies in adults have found it to be most useful in the selenium-depleted population, as there is a good correlation between whole blood GSHpx activity and selenium levels up to 1.27 µmol/L, beyond which selenium concentration continues to rise while GSHpx activity plateaus [185]. In neonates, serum selenium decreases from birth to 1 year of age, then steadily increases thereafter, which reflects increased requirements during the neonatal period, and possible dietary deficiency [186]. Breastfeeding has been shown to consistently result in higher selenium levels as compared to formula feeding or PN [171,187]. GSHpx activity, however, can be influenced by oxygen exposure and shows a very weak correlation with serum selenium in newborn infants, which limits its applicability, especially in premature neonates on supplemental oxygen [188].

#### 4.3.3. Selenium in Critical Illness

Serum selenium levels are known to decrease with systemic inflammation, most likely as a result of tissue redistribution rather than true deficiency [182]. Assessment of selenium status in critically ill children, therefore, has to take into account both the degree of inflammation and the nutritional status. An inverse correlation between CRP and serum selenium is only accurate in well-nourished children, while the relationship between nutritional status and serum selenium is best assessed in the absence of severe inflammation [189]. An examination of children enrolled in the CRISIS prevention trial also revealed that chronically ill children and those suffering from infection or sepsis are more likely to have depressed serum selenium levels [168]. Low serum selenium or an increased fraction of reduced GSHpx has also been associated with increased incidence of multi-organ failure in children admitted to the PICU [190]. Vice versa, an increase in serum selenium during critical illness has been associated with decreased ventilator dependence, ICU length of stay, and even mortality [191]. Low serum selenium and depressed GSHpx are associated with an increased incidence of infections in children with burn injuries [192]. However, it remains unclear whether low serum selenium directly impacts clinical outcomes, or whether it is secondary to a higher requirement for selenium and protection against oxidative stress in more severe illnesses. 

Premature infants, especially those with VLBW, are especially at risk for selenium deficiency. In Japanese low-birthweight premature infants, the lack of selenium supplementation in formula led to a steep decline in serum selenium levels, and especially erythrocyte selenium levels, indicating a state of true deficiency [170]. Furthermore, baseline serum selenium and GSHpx activity both correlate with birthweight in preterm neonates admitted to the ICU, which highlights the relationship between selenium status and intrauterine growth [193]. Serum selenium levels measured at 6 weeks were severely low, and almost half dropped below the detection limit in those who were dependent on PN and not being given selenium supplementation. Breastmilk proves to be the best source of selenium for preterm infants compared to formula or PN. However, breastfed preterm infants still fail to match the serum selenium concentration of term infants at 6 weeks, possibly as a result of lower total selenium storage at birth due to shorter gestation and lower selenium transfer from the mother [187]. There is also evidence that serum selenium concentrations at 1 month of age inversely correlate with oxygen-dependent days as well as the incidence of chronic lung disease in VLBW infants admitted to the ICU [194]. In this population, GSHpx activity shows a poor correlation with selenium levels and is less useful in the assessment of selenium status. 

Selenium supplementation has been extensively studied in critically ill adults with mixed results [182]. Supplementation of critically ill children with selenium as a part of the CRISIS prevention trial has not found an advantage in reducing the rate of nosocomial infection [174]. In neonates, selenium supplementation has gained attention mostly in preterm infants due to the lack of selenium in PN and their poor selenium storage as mentioned above. Parenteral selenium supplementation at the dose of 1.34 μg/kg per day is insufficient to raise the serum selenium levels in PN-dependent low-birthweight infants [195]. Parenteral selenium supplementation at the dose of 3 μg/kg per day maintains the serum levels but still fails to reach the levels seen in breastfed term infants [196]. It is interesting to note that there is a trend toward earlier recovery from bronchopulmonary dysplasia (BPD) and a lower incidence of sepsis in these two studies, although the low numbers of participants were insufficient to power these analyses. Alternatively, oral selenium supplementation with 5 or 10 μg daily can also raise serum selenium levels in VLBW premature infants [197,198]. Selenium supplementation at the dose of 10 μg/day results in a lower incidence of sepsis, but no difference in all-cause mortality. Selenium supplementation, therefore, might be useful in selenium-depleted VLBW neonates in the prevention of severe infection. However, its utility beyond this clinical scenario remains unproven. 

### 4.4. Copper

#### 4.4.1. Introduction

Copper is the essential co-factor for enzymes involved in the electron transport chain, neurotransmitter synthesis, protection against oxidative injuries, and iron transportation [199]. As a result, copper deficiency results in a series of characteristic hematologic and neurologic disorders. In infants, symptoms of copper deficiency include psychomotor retardation, neutropenia, iron therapy-resistant sideroblastic anemia, hepatosplenomegaly, and osteopathic changes among others [199]. 

#### 4.4.2. Assessment

Copper is mostly stored in the liver, which accounts for almost 50% of the total copper content in the newborn [199]. More than 95% of the copper in circulation is carried in ceruloplasmin, a ferroxidase enzyme that is synthesized in the liver and also plays a key role in iron metabolism [200]. Measurement of serum copper and ceruloplasmin is the most common method to assess for copper status, despite ceruloplasmin being a positive acute phase reactant. Additional assessment of copper status can also be achieved by measuring the activity of erythrocyte copper-zinc superoxide dismutase (SOD), which has been shown to be a sensitive indicator of copper functional status [201]. In infants, serum concentrations of copper and ceruloplasmin gradually increase in the postnatal period, reaching adult levels between 1 and 6 years of age [199,202]. Interestingly, there seems to be no correlation between the type of enteral feeding and serum copper levels [171]. There is, however, an inverse relationship between enteral zinc supplementation and serum markers of copper status, possibly as a result of the inhibitory effects of enteral zinc on copper absorption [203,204,205].

#### 4.4.3. Copper in Critical Illness

Copper levels are expected to rise in the face of systemic inflammation or infection since ceruloplasmin is a positive acute phase reactant. However, the increase in serum copper levels did not achieve statistical significance as compared to healthy controls in retrospective reviews of critically ill children and neonates admitted to a PICU and NICU, respectively [144,145]. Premature infants have significantly lower cord blood ceruloplasmin levels compared to term infants due to a marked acceleration in copper deposition in the fetus during the third trimester of pregnancy [198]. The ceruloplasmin levels continue to rise through the postnatal period and bear no relationship with the type of enteral feeding, or whether the infant was fed breastmilk or formula [206]. Interestingly, the erythrocyte SOD in VLBW infants does show a correlation with dietary copper intake and thus could be a more sensitive method to assess for functional copper status in this population [207]. The reduction in hepatobiliary flow, which constitutes the main route for copper excretion, results in a further increase in the serum concentrations of copper and ceruloplasmin in critically ill or PN-dependent VLBW infants [208]. Copper supplementation in premature infants remains controversial, as oral supplementation is an ineffective method to raise serum copper levels and the benefits of such therapy are still unproven [209,210]. 

## 5. Conclusions

Assessment of the micronutrient status in children presents a diagnostic challenge due to various reasons, including age-dependent metabolic demands, susceptibility to environmental influences, and changes in feeding methods and dietary intake among others. Plasma or serum levels of micronutrients are commonly measured, although they rarely reflect the total body storage and are strongly affected by tissue redistribution, especially in the setting of systemic inflammation or infection. Even though well-defined reference ranges exist for micronutrients, careful interpretation in the setting of critical illness is strongly advised. Micronutrient status in critically ill children should take into consideration both the presence of other inflammatory biomarkers, and the duration of inflammation. In addition, functional assessments of micronutrients by measuring their associated enzymes’ activities can provide a more accurate tool to evaluate their true status in children with critical illness. However, with the exception of certain micronutrients such as vitamin D, iron, and zinc, there remains a paucity of data on their status in infants or children admitted to the ICU. As long-term micronutrient deficiencies can lead to serious consequences later in life, recommendations on micronutrient supplements in neonatal and pediatric critical illness should be pursued based on large-cohort, longitudinal studies. 

## Figures and Tables

**Table 1:** Vitamin B complex and functions.

Vitamin	Name	Function and Enzyme Co-Factor
B_1_	Thiamine	Aerobic and carbohydrate metabolism
B_2_	Riboflavin	Oxidation-Reduction reactions: FAD and FMN
B_3_	Niacin	Oxidation-Reduction reactions: NAD and NADP
B_5_	Pantothenic acid	Acylation and acetylation: coenzyme A
B_6_	Pyridoxal Phosphate	Metabolism of proteins, carbohydrates, and fats
B_7_	Biotin	Carboxylase enzymes
B_9_	Folate	DNA and RBC synthesis
B_12_	Cobalamin	DNA, RBC, and myelin synthesis

Abbreviations: DNA, deoxyribonucleic acid; FAD, flavin adenine dinucleotide; FMN, flavin mononucleotide; NAD, nicotinamide adenine dinucleotide; NADP, nicotinamide adenine dinucleotide phosphate; RBC, red blood cells.

**Table 2:** Tests and their respective purposes of commonly used methods used to assess micronutrient status.

	Test	Purpose
**Vitamin B_1_**	Erythrocyte thiamine pyrophosphate	Quantification of thiamine pyrophosphate in erythrocytes
	Erythrocyte transketolase	Functional assessment of coenzyme activity
**Vitamin B_2_**	Erythrocyte glutathione reductase activity coefficient assay	Functional assessment of coenzyme activity
	Erythrocyte FAD	Measurement of the active form of riboflavin
**Vitamin B_3_**	Erythrocyte NAD and NADP	Measurement of active coenzymes of niacin
	Urinary 1-MN and 2-PYR	Measurement of niacin metabolites
**Vitamin B_5_**	Urine pantothenic acid	Direct quantification of vitamin B_5_
**Vitamin B_6_**	Plasma pyridoxal phosphate	Direct quantification of the active form of vitamin B_6_ in plasma
	Erythrocyte pyridoxal phosphate	Direct quantification of the active form of vitamin B_6_ in erythrocytes
	Plasma PA:(PL+PLP) ratio	Measurement of vitamin B_6_ catabolism in plasma
**Vitamin B_7_**	Urinary 3-HIA and 3-HIAc	Indirect measurement of biotin-dependent mitochondrial carboxylase activity
	Holo-PCC and holo-MCC	Quantification of vitamin B_7_-dependent enzymes in lymphocytes
**Vitamin B_9_**	Plasma 5-methyltetrahydrofolate	Measurement of the primary active form of folate
	Plasma homocysteine	Measurement of a precursor substrate for an enzyme that requires vitamin B_9_
**Vitamin B_12_**	Plasma cobalamin	Direct quantification of vitamin B_12_
	Plasma homocysteine	Measurement of a precursor substrate for an enzyme that requires vitamin B_12_
	Plasma MMA	Measurement of a precursor substrate for an enzyme that requires vitamin B_12_
	Urine MMA	Measurement of a precursor substrate for an enzyme that requires vitamin B_12_
**Vitamin C**	Plasma ascorbic acid	Direct quantification of vitamin C
**Vitamin A**	Plasma retinol	Direct quantification of one of the three forms of vitamin A
	Plasma retinol/RBP molar ratio	Measurement of proportion of a form of vitamin A and one of its circulating carrier proteins in plasma
	Relative-dose-response of retinol	Measurement of a form of vitamin A in response to its administration
	Changes in RBP following vitamin A administration	Measurement of one of the circulating carrier proteins of vitamin A in plasma in response to administration of vitamin A
	Fasting plasma retinyl esters	Direct quantification for vitamin A toxicity
**Vitamin D**	Plasma 25(OH)D	Direct quantification of free vitamin D metabolite
	Plasma bioavailable 25(OH)D	Direct quantification of free plus albumin-bound vitamin D metabolite
**Vitamin E**	Plasma alpha-tocopherol	Direct quantification of the major isoform of vitamin E (consider adjusting to cholesterol or total lipids in critically ill patients)
**Vitamin K**	Plasma phylloquinone	Direct quantification of the major form of vitamin K in plasma
	Coagulation function (INR and PT)	Functional assessment of vitamin K-dependent coagulation factors
	PIVKA-II and uOC	Measurement of undercarboxylated prothrombin and osteocalcin
**Iron**	Serum iron	Direct quantification of iron levels in serum
	Ferritin	Measurement of iron storage
	Transferrin/TIBC	Measurement of the capacity for iron transport
	Transferrin saturation	Quantification of the percentage of iron-bound transferrin
	ZnPP/H	Measurement of the proportion of protoporphyrin molecules associated with zinc vs. iron
**Calcium**	Serum total calcium	Direct quantification of total circulating calcium in serum
	Serum ionized calcium	Direct quantification of free circulating calcium in serum
**Magnesium**	Serum magnesium	Direct quantification of magnesium levels in serum
	Serum ionized magnesium	Direct quantification of ionized magnesium in serum
	24-hour urinary magnesium	Measurement of magnesium levels in urine
**Phosphorus**	Serum phosphate	Direct quantification of phosphate levels in serum
**Zinc**	Serum or plasma zinc	Direct quantification of zinc levels in serum or plasma
	Metallothionein	Measurement of zinc storage
	ALP	Functional assessment of a zinc-dependent enzyme
**Selenium**	Serum selenium	Direct quantification of selenium levels in serum
	GSHpx activity	Functional assessment of the main selenium-dependent enzyme
**Copper**	Serum copper	Direct quantification of copper levels in serum
	Ceruloplasmin	Measurement of the main protein for copper transport
	SOD activity	Functional assessment of a major copper-dependent enzyme

Abbreviations: 1-MN, 1-methylnicotinamide; 2-PYR, I-methyl-2-pyridone-5-carboxamide; 3-HIA, hydroxyisovaleric acid; 3-HIAc, hydroxyisovalerylcarnitine; 25(OH)D, 25-hydroxyvitamin D; ALP, alkaline phosphatase; FAD, flavin adenine dinucleotide; GSHpx, glutathione peroxidase; holo-MCC, 3-methylcrotonyl-CoA carboxylase; holo-PCC, propionyl-CoA carboxylase; INR, international normalized ratio; MMA, methylmalonic acid; PA, pyridoxic acid; PL, pyridoxal; PLP, pyridoxal 5’-phosphate; PIVKA-II, protein-induced in vitamin K absence-II; PT, prothrombin time; RBP, retinol-binding protein; SOD, superoxide dismutase; TIBC, total iron binding capacity; uOC, undercarboxylated osteocalcin; VDBP, vitamin D-binding protein; ZnPP/H, zinc protoporphyrin/heme ratio
